# Veteran-derived cerebral organoids display multifaceted pathological defects in studies on Gulf War Illness

**DOI:** 10.3389/fncel.2022.979652

**Published:** 2022-12-23

**Authors:** Philip L. Yates, Kendra Case, Xiaohuan Sun, Kimberly Sullivan, Peter W. Baas, Liang Qiang

**Affiliations:** ^1^Department of Neurobiology and Anatomy, Drexel University College of Medicine, Philadelphia, PA, United States; ^2^Department of Environmental Health, Boston University School of Public Health, Boston, MA, United States

**Keywords:** Gulf War Illness (GWI), veterans, human induced pluripotent stem cell (hiPSC), cerebral organoid, tau, microtubule, astrocyte reactivation, neurogenesis

## Abstract

Approximately 30% of the veterans who fought in the 1991 Gulf War (GW) suffer from a disease called Gulf War Illness (GWI), which encompasses a constellation of symptoms including cognitive deficits. A coalescence of evidence indicates that GWI was caused by low-level exposure to organophosphate pesticides and nerve agents in combination with physical stressors of the battlefield. Until recently, progress on mechanisms and therapy had been limited to rodent-based models. Using peripheral blood mononuclear cells from veterans with or without GWI, we recently developed a bank of human induced pluripotent stem cells that can be differentiated into a variety of cellular fates. With these cells, we have now generated cerebral organoids, which are three-dimensional multicellular structures that resemble the human brain. We established organoid cultures from two GW veterans, one with GWI and one without. Immunohistochemical analyses indicate that these organoids, when treated with a GW toxicant regimen consisting of the organophosphate diisopropyl fluorophosphate (a sarin analog) and cortisol (to mimic battlefield stress), display multiple indicators consistent with cognitive deficits, including increased astrocytic reactivity, enhanced phosphorylation of tau proteins, decreased microtubule stability, and impaired neurogenesis. Interestingly, some of these phenotypes were more pronounced in the organoids derived from the veteran with GWI, potentially reflecting a stronger response to the toxicants in some individuals compared to others. These results suggest that veteran-derived human cerebral organoids not only can be used as an innovative human model to uncover the cellular responses to GW toxicants but can also serve as a platform for developing personalized medicine approaches for the veterans.

## Introduction

Gulf War Illness (GWI) is a chronic multi-system disorder suffered by approximately 30% of the nearly 700,000 United States veterans deployed during the 1991 Gulf War (GW) (RAC, 2008, 2014; GWIRP, 2020). Prominent central nervous system (CNS) symptoms include memory deficits, slowed information processing speeds, poor attention, chronic headaches, and impaired sleep and mood (White et al., 2016; Janulewicz et al., 2017; Jeffrey et al., 2019). There is consensus in the field that GWI was caused by exposure to low-dose organophosphate pesticides and nerve agents in combination with physical stressors of the battlefield (Sullivan et al., 2003, 2018). These low-dose exposures implicate novel biological targets that are different than the classic cholinergic mechanism of the toxicants (Terry, 2012; Ross et al., 2013). The most prevalent hypothesis for the underlying etiology of GWI is a state of chronic neuroinflammation induced by the toxicant/stress exposure (Locker et al., 2017; Alshelh et al., 2020; Michalovicz et al., 2020). It is unknown, however, whether neuroinflammation is the primary cause of the cognitive deficits or whether neuroinflammation is upstream or downstream of other pathological mechanisms that are relevant to the symptoms.

A significant challenge in the GWI field has been the development of appropriate models for studying the underlying mechanisms of the disease and for testing potential therapies. To date, rodent models have demonstrated reactive astrogliosis (Zakirova et al., 2015; Madhu et al., 2019), changes in cytokines indicative of neuroinflammation (O’Callaghan et al., 2015; Locker et al., 2017; Koo et al., 2018; Cheng et al., 2020), and cognitive deficits that include memory decrements (Terry et al., 2012; Parihar et al., 2013; Hattiangady et al., 2014; Kodali et al., 2018), all of which persist months after toxicant exposure. Consistently, the full range of symptoms displayed by these animals depends upon inclusion with the toxicant regimen of either physical restraint of the animal or administration of corticosterone to mimic the battlefield stress component of GWI. Even so, rodents are not human, and many aspects of human pathology may not be accurately recapitulated in rodents. In addition, there is a long history of therapies for various diseases proving effective in rodents and yet failing in humans. For these reasons, we created a bank of human induced pluripotent stem cell (hiPSC) lines from GW veterans with or without GWI, so that these cells can be differentiated into various types of neurons, glia, and other cell types relevant to GWI symptoms (Qiang et al., 2017). Ideally, these cells can be used in experiments alongside the rodent models so that the advantages of each model complement one another.

In our first experimental work with these cell lines, we differentiated them into glutamatergic neurons and treated them with a GWI regimen of the sarin analog diisopropyl fluorophosphate (DFP) and cortisol (to mimic battlefield stress) (Yates et al., 2021). We found a number of cellular defects as a result of the regimen, many of which appeared to be worse in cells derived from veterans with GWI than without, which is consistent with the possibility that soldiers who acquired the disease may have been predisposed to the disease mechanisms underlying GWI (Yates et al., 2021). We observed reductions in microtubule acetylation and alterations in mitochondrial health, dynamics, and transport, all of which are consistent with earlier work on cultured rodent neurons exposed to GW toxicant regimens (Middlemore-Risher et al., 2011; Gao et al., 2016; Rao et al., 2017; Yates et al., 2021). We also observed reductions in neuronal activity and elevations in the levels and pathological phosphorylation of tau, a microtubule-associated protein that goes awry in a number of neurodegenerative disorders that involve cognitive deficits (Bodea et al., 2016; Kanaan et al., 2016; Wang and Mandelkow, 2016; Yates et al., 2021). The tau results aligned well with observations on a corresponding rat model for GWI, which showed both behavioral deficits and tau pathology. The tau defects in the rat model occurred specifically in the CA3 region of the hippocampus, which correlates with observations on veterans with GWI (Chao et al., 2014; Chao and Zhang, 2018; Yates et al., 2021).

These studies to date were conducted on two-dimensional neuronal cultures in which the cells were adhered to a glass substrate. While a powerful model system, these cultures have limitations, such as the lack of higher order brain structure and the lack of key cellular components of the brain, such as astrocytes, that might be relevant to GWI pathogenesis. To overcome these shortcomings, we sought to generate three-dimensional human cerebral organoids, also called mini-brains, from the hiPSC lines. Compared to two-dimensional neuronal cultures, cerebral organoids are hierarchically-organized multicellular structures with sophisticated interstitial compartments. These organoids, which include multiple cell types in an orderly and physiological environment, recapitulate much of the anatomy of a human brain, including neuroepithelial loops of neuronal progenitor cells (NPCs) that are reminiscent of ventricular zones, as well as the multiple layers of the developing cortex (Lancaster et al., 2013; Luo et al., 2016; Qian et al., 2016). Here, in the first studies using organoid technology to model GWI, we generated human forebrain cerebral organoids from one cell line each from veterans with or without GWI. After GW toxicant exposure, immunohistochemical analyses were performed to examine markers for inflammation, tauopathy, microtubule stability, and neurogenesis, all of which are potentially relevant to the cognitive deficits suffered by veterans with GWI.

## Materials and methods

### hiPSC culture

Human induced pluripotent stem cell lines were generated from peripheral blood mononuclear cells (PBMCs) from GW veterans with or without GWI, according to the Kansas case definition (Steele, 2000), and banked in the Boston Biorepository, Recruitment and Integrative Network (BBRAIN) for GWI (Qiang et al., 2017). The hiPSCs were previously validated for their pluripotency *via* morphology, karyotype, immunostaining for pluripotency markers, and three-germ layer differentiation (Yates et al., 2021). All hiPSCs and organoids were cultured in 5% CO2 at 37°C. hiPSCs were cultured as previously reported (Yates et al., 2021) in mTeSR1 (Stem Cell Technologies, Vancouver, BC, Canada, 85857) on embryonic stem cell qualified Matrigel (Corning, Corning, NY, United States, 354277) diluted 1:75 in cold DMEM/F12 (Gibco, Waltham, MA, United States, 11330-032), and passaged weekly at 80% confluency using Collagenase Type IV (Stem Cell Technologies, Vancouver, BC, Canada, 07909) or ReLeSR (Stem Cell Technologies, Vancouver, BC, Canada, 05872) with 10 μM ROCK inhibitor Y-27632 (Tocris, Bristol, United Kingdom, 1254) after selectively removing areas of spontaneous differentiation.

### Generation of hiPSC-derived cerebral organoids and GW toxicant treatment

Two representative hiPSC lines from one healthy GW veteran (control) and one veteran with GWI (Steele, 2000) were used to generate dorsal forebrain cerebral organoids *via* previously established protocols (Lancaster et al., 2013; Lancaster and Knoblich, 2014a) with adaptations. Briefly, hiPSC colonies were dissociated in ReLeSR (Stem Cell Technologies, Vancouver, BC, Canada, 05872) for 5 min at 37°C, after which cells were collected then resuspended in mTeSR Plus (Stem Cell Technologies, Vancouver, BC, Canada, 100-0276), 10 μM SB-4321542 at 20 ng/ml (Tocris, Bristol, United Kingdom, 1614), and the ROCK inhibitor Y-27632 at 10 μM (Tocris, Bristol, United Kingdom, 1254) on ultra-low adherence plates (Corning, Corning, NY, United States, 3471) for embryoid body (EB) formation. The medium was replaced on day 3 and supplemented with dorsomorphin (Sigma-Aldrich, St. Louis, MO, United States, P5499) and SB-4321542. On day 6 the medium was replaced with Neurobasal Differentiation Media (NBM), consisting of Neurobasal-A media (Life Technologies, Carlsbad, CA, United States, cat. no. 10888022) with B27 supplement without vitamin A (Life Technologies, Carlsbad, CA, United States, 12587010) at 2% of the total volume and GlutaMax (Life Technologies, Carlsbad, CA, United States, cat. no. 35050–061) at 1% of the total volume. NBM was supplemented with 20 μg/ml basic fibroblast growth factor (bFGF) (PeproTech, Cranberry, NJ, United States, 100-18B) and 20 μg/ml epidermal growth factor (EGF) (PeproTech, Cranberry, NJ, United States, AF-100-115) from days 6–24. On day 11 the plates were moved to an orbital shaker (Infors HT Celltron, Annapolis Juntion, MD, United States, I69222) at 37°C set at 65 revolutions per minute. From day 26 onward the NBM was supplemented with 20 ng/ml brain-derived neurotrophic factor (BDNF) (PeproTech, Cranberry, NJ, United States, 450-02) and 20 ng/ml neurotrophin 3 (NT3) (PeproTech, Cranberry, NJ, United States, 450-03). The medium was changed every 2–3 days throughout the duration of organoid generation.

A total of 2.5 months old organoids were exposed to a GW toxicant regimen adapted from established rodent models (O’Callaghan et al., 2015; Locker et al., 2017) and modified from studies on rat and human two-dimensional neuronal cultures (Rao et al., 2017; Yates et al., 2021) in order to prolong the exposure for cerebral organoids. The toxicant regimen consisted of exposing the organoids to 7 days of 2 μM hydrocortisone (Cortisol) (Sigma-Aldrich, St. Louis, MO, United States, H0888), dissolved in 10% ethanol in ddH2O, followed by 4 days of 2 μM Cortisol plus 200 nM diisopropyl fluorophosphate (DFP) (Sigma-Aldrich, St. Louis, MO, United States, D0879), dissolved in isopropanol, followed by 7 days of washout.

### Quantitative immunohistochemical analyses

Gulf War toxicant treated organoids were fixed in pre-warmed 4% paraformaldehyde (Electron Microscopy Sciences, Hatfield, PA, United States, 19202) in 0.1 M phosphate buffered saline (PBS) for 4 h on a rotator at 4°C, and then changed to a 30% sucrose solution in PBS overnight. The next day, 3–5 organoids were placed in a Tissue-Tek cryomold (Sakura Tissue-Tek Cryomold, Torrance, CA, United States, 4566), embedded in M-1 Shandon embedding matrix (Thermo Scientific, Waltham, MA, United States, 1310), and frozen in isobutane cooled to –40°C with dry ice. Frozen organoids were sectioned at 20 μm thickness on a HM500 OM Series Microm microtome cryostat at –20°C onto positively supercharged microscope slides (Fisher, Waltham, MA, United States, 12-550-16). Slides were dried overnight at room temperature and then stored at 4°C.

For immunohistochemical experiments, slides were rehydrated in PBS before permeabilization with 0.1% Triton X-100 (Sigma-Aldrich, St. Louis, MO, United States, X100) diluted in PBS for 10 min at room temperature, and then blocked with 10% goat serum (Jackson ImmunoResearch, Westgrove, PA, United States, 005-000-121) or 10% donkey serum (Jackson ImmunoResearch, Westgrove, PA, United States, 017-000-121) diluted in PBS for 1 h at room temperature. Primary antibodies used were mouse anti-Tau13 (1:4000; donation from Nicholas Kanaan, Michigan State University), rabbit anti-tau ps422 (1:200; Abcam, Cambridge, United Kingdom, ab79415), chicken anti-Tbr1 (1:300; Millipore-Sigma AB2261), rabbit anti-SATB2 (1:500; Abcam, Cambridge, United Kingdom, ab34735), goat anti-GFAP (1:3000; Abcam, Cambridge, United Kingdom, ab53554), rabbit anti-S100ß (1:1000; Abcam, Cambridge, United Kingdom, ab41548), mouse anti-NeuN (1:500; Abcam, Cambridge, United Kingdom, 104224), goat anti-Sox2 (1:1000; R&D Systems, Minneapolis, MN, United States, AF2018), rabbit anti-Sox2 (1:500; Millipore-Sigma, Burlington, MA, United States, AB5603), rabbit anti-ßIII-tubulin (1:2000; BioLegend, San Diego, CA, United States, 802001), mouse anti-acetylated tubulin (1:8000; Sigma-Aldrich, St. Louis, MO, United States, T6793). Appropriate goat or donkey secondary antibodies conjugated to Alexa Fluor 488, 555, and 647 were used at 1:1000 dilutions. Images were acquired on a Zeiss AxioObserver Z1 inverted microscope equipped with a Zeiss 20X/0.8 Plan-Apochromat air objective and AxioCam 506 mono camera using the Zeiss Zen Blue software (Zeiss, Oberkochen, Germany). All images from each replicate were acquired with identical exposure settings under conditions of no pixel saturation and minimal bleaching during image acquisition. Fluorescence intensity was quantified using ImageJ version 2.3.0/1.53q by measuring the arbitrary fluorescence units per organoid area.

These experiments were performed in approximately 20 different organoids derived from three different batches in the control line and approximately 30 different organoids derived from five different batches in the case line. 20x images were taken at various areas spanning the organoid and each image was treated as a separate datapoint. The neuroepithelial loops were quantified by the number of loops per area, as well as the areas of the lumen and the proliferative zone. To account for any differences in the size of the neuroepithelial loops between organoids, the percentages of area occupied by the lumen and the proliferative zone were calculated relative to the total neuroepithelial loop area. Sox2 and NeuN cell counts were quantified using ImageJ. To examine the number of loops per area, the size of neuroepithelial loops, and Sox2 and NeuN cell counts, tile scans of the complete organoid section were used instead of 20x single images. Only sections with no significant rips or central necrosis were used. The whole section was used to mitigate any size differences between the organoids, as well as any neuroepithelial loop location and/or clustering biases. These whole sections were used for the NeuN and Sox2 counts to determine total distribution across the organoid section instead of potential biases using single images.

While a similar number of organoids were originally stained, the integrity of the entire section dictated which ones could be analyzed. This accounts for the lower number of datapoints for neurogenesis. Additionally, only sections containing at least one neuroepithelial loop were used for the analyses of all experimental parameters except total Sox2 and NeuN cell counts, further narrowing down the data pool.

### Experimental design and statistical analyses

All data analyses were conducted blind and presented as mean ± SEM relative to vehicle per line. At least three independent repeats were conducted for each experiment. Data were arranged in Microsoft Excel before statistical analyses were performed using Prism 9 (GraphPad). All data sets were tested for normality using Shapiro-Wilk’s test, and outliers were excluded using the GraphPad ROUT method (a three-step process that includes a robust non-linear regression, the residuals were analyzed for outliers, and an ordinary least-squares regression was performed on the remaining data) with a coefficient of Q = 0.1% set at a strict threshold to remove definitive outliers. The statistical test used was one-way ANOVA (Kruskal-Wallis test for data not normally distributed). An alpha of *p* < 0.05 was considered statistically significant.

## Results

### Generation of GW veteran-derived cerebral organoids

**FIGURE 1:**
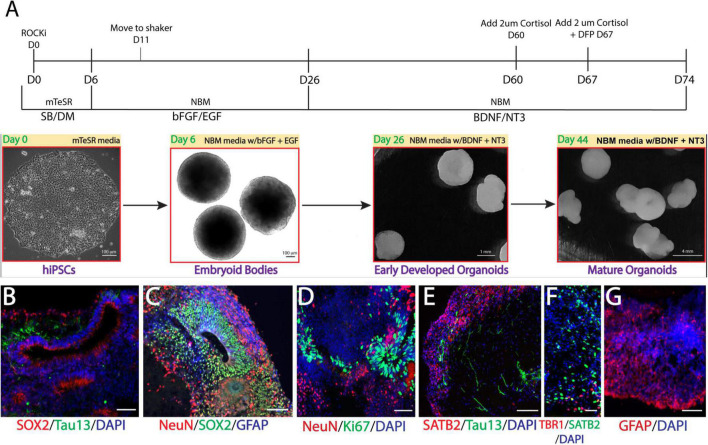
Generation of Gulf War (GW) veteran-derived forebrain cerebral organoids. **(A)** Schematic of the procedure used to generate forebrain cerebral organoids [Dorsomorphin (DM), SB-4321542 (SB), basic fibroblast growth factor (bFGF), epidermal growth factor (EGF), brain-derived neurotrophic factor (BDNF), neurotrophin 3 (NT3), Neurobasal Differentiation Media (NBM)]. **(B–D)**. Immunofluorescence images of forebrain cerebral organoids stained for the proliferative markers Sox2 and Ki67, neuronal markers tau and NeuN, the astrocytic marker glial fibrillary acidic protein (GFAP), and 4, 6-diamino-2-phenylindol (DAPI) nuclei. **(E, F)**. Images stained for the cortical layer markers SATB2, Tbr1, tau, and DAPI. **(G)** Image shows astrocyte GFAP staining. Scale bars **(B–G)**, 50 μm.

We recently generated multiple lines of hiPSCs reprogramed from PBMCs donated by veterans with or without GWI (Qiang et al., 2017) and banked them in the Boston Biorepository, Recruitment and Integrated Network for GWI (BBRAIN) (Keating et al., 2021). Previously, we validated and differentiated these hiPSC lines into forebrain glutamatergic neurons (Yates et al., 2021). Here, we selected two representative lines, one control line and one case line, to generate forebrain cerebral organoids. The schematic in Figure 1A diagrams the method of creating forebrain cerebral organoids. Immunofluorescence results show that the organoids cultured for 2.5 months recapitulated many features of the anatomy of the human brain, including forming neuroepithelial loops expressing the neuronal progenitor cell (NPC) marker Sox2 and the proliferation marker Ki67, whereas tau and NeuN positive neurons were located outside of the neuroepithelial loops, which is reminiscent of a ventricular zone and a cortical plate (Figures 1B–D). The cortical plate displayed early-born deep-layer Tbr1 positive neurons and SATB2 positive late-born more superficial neurons (Figures 1E, F). The organoids were also composed of GFAP-positive cells indicative of radial glial cells at younger ages and astrocytes at older ages (Figures 1C, G). Only a very small number of oligodendrocytes and no microglia were observed in our current organoid models.

### GW toxicants produce tau pathology in cerebral organoids

**FIGURE 2:**
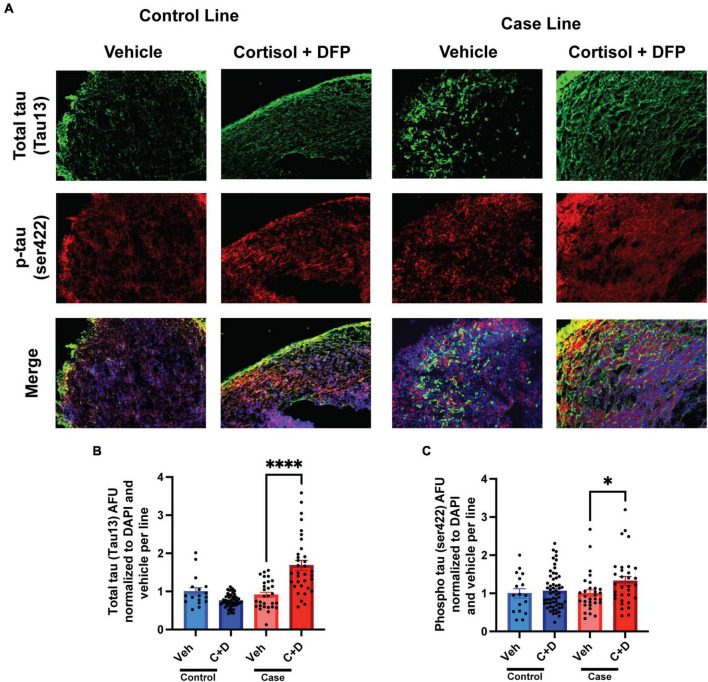
Gulf War (GW) toxicant regimen produces tau pathology in forebrain cerebral organoids. **(A)** Representative immunofluorescence images from one control line and one case line exposed to 2 μM cortisol plus 200 nM DFP show staining for total tau (green) and phosphorylated tau (red) and 4, 6-diamino-2-phenylindol (DAPI) (blue). **(B,C)** The graphs show mean arbitrary fluorescence units (AFU) ± SEM normalized to DAPI per line. Total tau increased in the case line by 77.36% (0.7736 ± 0.1412); ps422 increased in the case line only by 32.5% (0.3250 ± 0.1164). *p < 0.05, ^****^p < 0.0001.

2.5 months-old forebrain cerebral organoids from one representative control line and one representative case line were exposed to a GW toxicant regimen composed of the human stress hormone cortisol plus the sarin surrogate DFP. We first examined the effects of the toxicant regimen on tau expression levels in these organoids *via* quantitative immunofluorescence because tau pathology is involved in numerous neurological disorders, and evidence indicates that organophosphate exposure alters cytoskeletal homeostasis (Abou-Donia, 1995; Abou-Donia et al., 2017, 2020). We found that the GW toxicant regimen increased total tau levels (Tau13 antibody) selectively in the case line by 77.36% relative to vehicle exposed organoids (Figures 2A, B and Supplementary Table 1). Furthermore, phosphorylation at Ser422 in the tau protein, which is often identified as one of the early pathological phosphorylation sites in various tauopathies (Guillozet-Bongaarts et al., 2006; Vana et al., 2011; Collin et al., 2014; Tiernan et al., 2016), increased significantly by 32.50% only in the case line (Figures 2A, C and Supplementary Table 1). There was also a potential decrease (which did not achieve statistical significance) in total tau levels in the control line (*p* = 0.0657) when exposed to the GW toxicant regimen.

### Reduced microtubule acetylation in cerebral organoids exposed to GW toxicants

**FIGURE 3:**
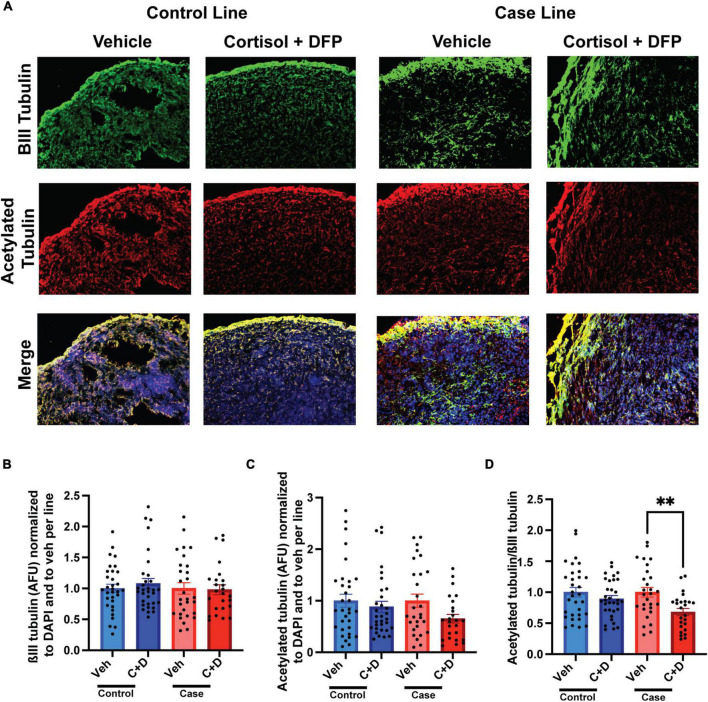
Reduced microtubule acetylation in forebrain cerebral organoids exposed to Gulf War (GW) toxicants. **(A)** Representative immunofluorescence images from one control line and one case line exposed to 2 μM cortisol plus 200 nM diisopropyl fluorophosphate (DFP) show staining for ßIII-tubulin (green) acetylated tubulin (red), and 4, 6-diamino-2-phenylindole (DAPI) (blue). **(B)** The graph shows mean arbitrary fluorescence units (AFU) ± SEM of ßIII tubulin normalized to DAPI per line and to vehicle per line. **(C)** The graph shows mean AFU ± SEM of acetylated tubulin normalized to DAPI per line and to vehicle per line. **(D)** The graph shows the ratio of acetylated tubulin ßIII normalized to DAPI per line and to vehicle per line. The acetylated tubulin/ßIII tubulin ratio decreased in the case line by 31.95% (0.3195 ± 0.0582). ^**^p < 0.01.

We previously documented in primary rat neurons and hiPSC-derived neurons that GW toxicant exposure reduces microtubule acetylation (Rao et al., 2017; Yates et al., 2021), which can be used as a marker for the stable domain of microtubules (Baas et al., 2016). Consistently, in our present studies on organoids, we found a significant reduction in the ratio of microtubule acetylation to total neuronal ßIII-tubulin only in the case line by 31.95% relative to vehicle exposed organoids (Figures 3A–D and Supplementary Table 2).

### Enhanced astrocytic reactivity in cerebral organoids exposed to GW toxicants

**FIGURE 4:**
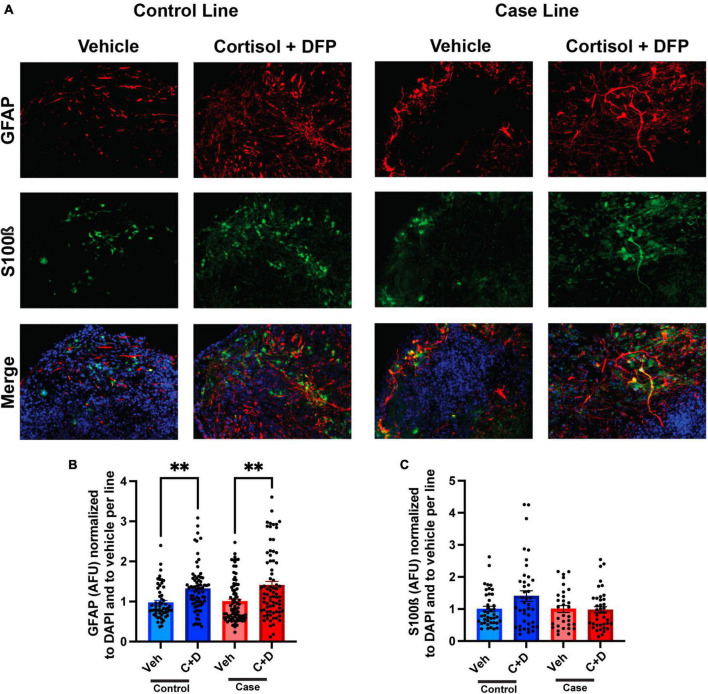
Reactive astrogliosis in forebrain cerebral organoids exposed to a Gulf War (GW) toxicant regimen. **(A)** Representative immunofluorescence images from one control line and one case line exposed to 2 μM cortisol plus 200 nM diisopropyl fluorophosphate (DFP) show staining for the astrocyte maker glial fibrillary acidic protein (GFAP) (red), S100ß (green), and 4, 6-diamino-2-phenylindole (DAPI) (blue). **(B)** The graph shows mean arbitrary fluorescence units (AFU) ± SEM normalized to DAPI per line; GFAP increased in the control line by 34.934% (0.3493 ± 0.0704) and in the case line by 40.5% (0.4050 ± 0.0960). **(C)** The graph shows mean AFU ± SEM of S100ß normalized to DAPI per line. ^**^p < 0.01.

The predominant hypothesis in the GWI field is that toxicant exposure induces a chronic neuroinflammatory state that leads to persistent cognitive and other chronic health symptoms (Locker et al., 2017; Alshelh et al., 2020; Michalovicz et al., 2020). Since astrocyte reactivity plays a significant role in neuroinflammation (Colombo and Farina, 2016; Liddelow and Barres, 2017), we sought to examine whether toxicant-induced astrocyte activation occurs in the forebrain cerebral organoids. In the present studies on organoids, the GW toxicant regimen increased fluorescence staining intensity for the astrocytic marker glial fibrillary acidic protein (GFAP) in both the control and case lines, with elevations of 34.93 and 40.50%, respectively (Figures 4A, B and Supplementary Table 3). GFAP levels have been used to assess the reactivation state of astrocytes, with increased levels indicating more inflammation (Liddelow and Barres, 2017). S100ß was used as a marker for mature astrocytes and to show that GFAP is not restricted to radial glia at these timepoints (Figures 4A, C).

### GW toxicants alter neurogenesis in cerebral organoids

**FIGURE 5:**
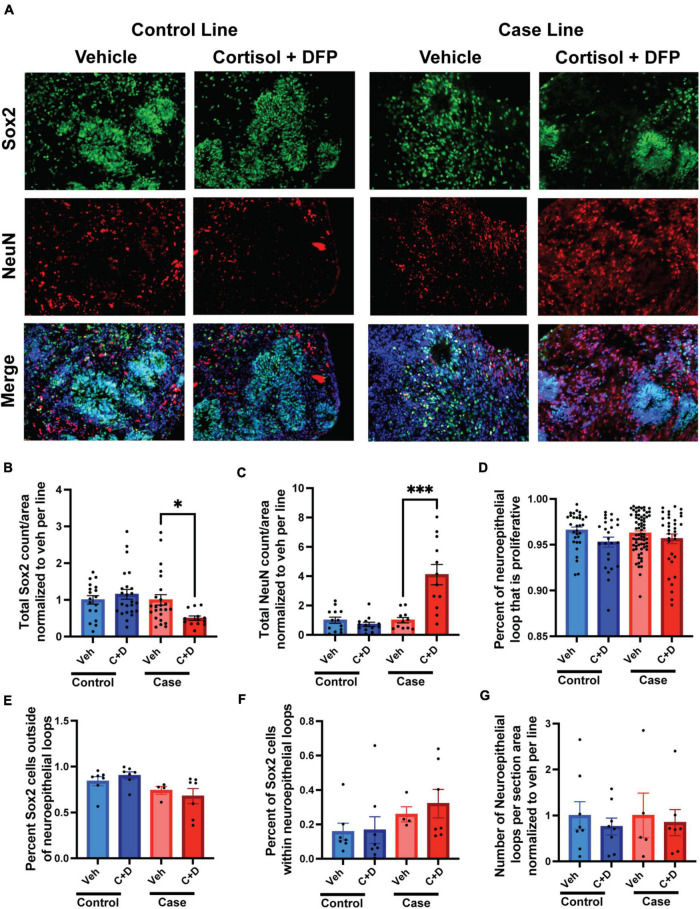
Gulf War (GW) toxicant exposure alters neurogenesis in forebrain cerebral organoids. **(A)** Representative immunofluorescence images from one control line and one case line exposed to 2 μM cortisol plus 200 nM diisopropyl fluorophosphate (DFP) show staining for the neuroprogenitor cell marker Sox2 (green), the neuronal marker NeuN (red), and 4, 6-diamino-2-phenylindole (DAPI) (blue). **(B,C)** These graphs show the counts of Sox2 cells decreased in the case line by 53.77% (0.5377 ± 0.0595) and the counts of NeuN cells increased in the case line by 310% (3.1 ± 0.6912) across the organoid sections normalized to vehicle per line. **(D)** This graph shows the size of the progenitor zone in the control line and case line, although there is no significant difference. **(E,F)** These graphs show the distribution of Sox2 positive cells associated or non-associated with a neuroepithelial loop. **(G)** This graph shows the number of neuroepithelial loops per organoid section normalized to vehicle per line. *p < 0.05, ^***^p < 0.001.

Memory complaints are one of the most common symptoms of veterans with GWI. Although primarily a developmental phenomenon, studies have shown that neurogenesis persists in the adult brain and is important for continued learning, memory, and cognition throughout life, and defects in adult neurogenesis can negatively impact these fundamental tasks (Alam et al., 2018; Berdugo-Vega et al., 2020; Babcock et al., 2021). Moreover, studies have shown that GW toxicants impair neurogenesis in adult rats and correspond to learning, memory, and cognitive deficits (Parihar et al., 2013; Kodali et al., 2018). Due to their ability to mimic neurodevelopment, cerebral organoids can be used as an appropriate model to study human neurogenesis. In the present study, we examined the effects of our GW toxicant regimen on neurogenesis in forebrain cerebral organoids. We found the GW toxicant regimen affected the ratio of progenitor cells to neurons only in the case line, with the overall amount of Sox2 positive cells reduced by 53.77% and the overall amount of NeuN positive cells increased by 310% (Figures 5A–C and Supplementary Table 4). The area of the proliferation zone, which is a belt-like structure composed of Sox2/Ki67 positive cells, can be used to evaluate how robust neurogenesis is maintained in human cerebral organoids (Lancaster and Knoblich, 2014a; Arber et al., 2021). We detected that the area of the proliferation zone in the neuroepithelial loop was slightly reduced by 1.78% trending toward significance (*p* = 0.0878) in the control organoids exposed to the toxicant regimen compared to the vehicle treated controls (Figure 5D). However, we did not detect any differences in the ratio of progenitor cells associated or non-associated with a neuroepithelial loop (Figures 5E–F). Finally, we found no difference in the number of neuroepithelial loops per organoid section area (Figure 5G).

## Discussion

The present studies are the first to use hiPSC-derived cerebral organoids to investigate cellular alterations in GWI. Rather than differentiating hiPSCs on an adherent substrate, culturing them in an ultralow attachment dish with a combination of spontaneous and directed differentiation using temporally controlled applications of various growth factors, patterning modules, as well as activation and deactivation of relevant signaling pathways, allows the cells to self-organize into a complex three-dimensional structure. As they grow and become more mature, the organoids recapitulate essential features of human brain anatomy, including ventricle-like proliferation zones and the layers of the developing cortex. As they mature, the organoids consist of a rich combination of astrocytes and neurons that interact in a three-dimensional environment, thus earning them the name mini-brains (Lancaster et al., 2013; Lancaster and Knoblich, 2014b). This environment encourages development of neuron-glia networks that allow for better modeling of human brain features, such as the differential expression of human tau isoforms that are not well-represented in the rodent brain (Hernandez et al., 2019, 2020). Studies have taken advantage of this unique cell culture system to model neurodevelopmental and neurodegenerative diseases using patient-specific cells to generate cerebral organoids with distinct brain regions, such as dorsal and ventral forebrain, midbrain, hypothalamus, and hippocampal organoids, using established protocols (Kofman et al., 2022).

In the present studies using cerebral organoids to model GWI, we used immunohistochemistry to evaluate tauopathy, microtubule stability, astrocytic reactivation, and neurogenesis after exposure to our GW toxicant regimen, as these cellular processes have been documented as important components of GWI pathology in studies on primary culture, animals, and GW veterans (Gao et al., 2016; Locker et al., 2017; Alshelh et al., 2020; Yates et al., 2021). We found that the regimen increased the levels of total tau and early pathologically phosphorylated tau at Ser422 in the case line. The Ser422 phosphorylation site is prominent in early stages of Alzheimer’s disease and other tauopathies, thus making it relevant to tau pathology onset and progression (Guillozet-Bongaarts et al., 2006; Vana et al., 2011; Collin et al., 2014; Tiernan et al., 2016). Consistent with the tau pathologies, a reduction in microtubule acetylation was identified only in the case line. The case line also exhibited robust changes in neurogenesis, with the decreased number of Sox2 positive neural progenitor cells and increased number of NeuN positive neurons suggesting premature cortical neural differentiation and maturation. This phenomenon has been associated with accelerated aging in some neurodegenerative disorders (Alam et al., 2018; Arber et al., 2021; Babcock et al., 2021), and could indicate that an accelerated aging process is implicated in the disease mechanisms of GWI. These individual differences in the case line in multiple cellular parameters relevant to GWI symptoms suggest that the case line might be more sensitive to GW toxicant exposure, and hence that veterans who developed GWI might be more vulnerable to develop these pathologies than their similarly exposed but healthy colleagues.

Interestingly, increased astrocyte reactivity, which is indicative of neuroinflammation (Colombo and Farina, 2016; Liddelow and Barres, 2017), was identified in the organoids derived from both lines. Levels of GFAP were used as a marker for activated astrocytes because greater levels of GFAP are associated with greater reactivity of astrocytes. Our results suggest that GW toxicant exposure enhanced astrocyte reactivity. This is consistent with a large body of work pointing to neuroinflammation as a driver of GWI pathology (Michalovicz et al., 2020), and validate the forebrain cerebral organoids as a suitable model to study GWI and the cellular interactions that contribute to increased neuroinflammation upon toxicant exposure. While neuroinflammation would be the most common expectation in the GWI field, the other phenomena we identified were every bit as robust. It should also be noted that microglia and oligodendrocytes were almost absent from our current cerebral organoids, indicating that the increase in astrocytic reactivity is likely astrocyte autonomous, or the result of crosstalk between neurons and astrocytes.

One important aspect of GWI research is to understand why some soldiers acquired the disease and others did not, despite being similarly exposed. This may be due to genetic predispositions or epigenetic variations, which cannot be explored in rodents with an identical genetic background. To date, we have observed both here and in our previous paper (Yates et al., 2021), several indications that the neurons and organoids from veterans with GWI are more sensitive to the GW toxicant regimen than counterparts from veterans without GWI, although both studies lack sufficient statistical power to draw strong conclusions on this point. The present studies are proof-of-concept that neuropathological effects consistent with GWI symptoms are elicited in human-derived organoid models and suggest that future work of this kind involving more veterans will significantly extend the knowledge obtained from animal and human studies. For example, we are interested in examining hiPSC lines for potential polymorphisms in paraoxonase-1 (PON1), a glycoprotein functionally associated with lipid metabolism, related to protection from low-level nerve agent exposures in GWI (Salazar et al., 2021; Haley et al., 2022).

The use of cerebral organoids fills a gap in the cadre of tools available to study GWI. Rodent models are important because they provide crucial behavioral and systems-level insights into the mechanisms of the disease, but a human-specific model is needed alongside the rodent models. This is because not all features of human disease are well-represented in rodents, and indeed there have been many failures over the years in clinical trials for neurodegenerative diseases of treatments that had very promising results in rodents. Brain organoids with distinct region identities, such as dorsal or ventral forebrain, midbrain, hippocampus, and hypothalamus, can be developed using established protocols (Kofman et al., 2022). These organoids with various brain region identities will be valuable for GWI studies since the disease might afflict multiple brain regions.

The organoid technology is among the most cutting-edge in the biomedical sciences and continues to improve rapidly, but still has critical limitations. For example, the organoids vary in size and their maturity remains undefined, and this hinders their applications in modeling age-related disorders. Efforts are underway by various research teams to reduce the variability and improve the reproducibility of organoids by restricting the type and number of originating cells, standardizing the reagents and procedures of the culture process and controlling the patterning and maturation strategies (Kofman et al., 2022). The application of defined biomaterials combined with 3D printing technology has also increased the reproducibility of the organoids (Kofman et al., 2022). In order to advance the maturity of organoid development, such strategies were evaluated as prolonged culture protocols that promote cell survival, growth and differentiation (Kofman et al., 2022); assembloid cultures that incorporate endothelium (Kofman et al., 2022); and slice cultures of the organoids with air-liquid interface, which promotes neuronal differentiation, axonal growth and synaptic formation (Giandomenico et al., 2019; Qian et al., 2020). To develop a GWI organoid model with more cellular complexity, we are currently aiming to assemble a more complex mini-brain model by including missing cellular components in the CNS, such as microglia, oligodendrocytes, microvascular endothelial cells and ependymal cells. Application of these advanced CNS assembloids will provide a more physiological and intricate *in vitro* 3D human CNS model for probing complex cell-cell interactions implicated in GWI. Moreover, organoids from the veteran-derived hiPSCs can also be created to represent other organ systems relevant to GWI symptoms, such as the intestines.

Taken together, the current studies and results provide the GWI field with a powerful new tool for elucidating the cellular etiology of the disease and testing potential therapies. In addition, these studies set the stage for realizing a personalized medicine approach for individualized treatment of veterans with GWI, which is important because different veterans were exposed to different toxicant regimens and because each veteran went into the battlefield with different genetic and epigenetic dispositions for neurodegenerative pathways. In theory, brain organoids derived from each veteran can be preclinically screened for compounds or reagents that ameliorate cellular pathologies related to the disease and which might be particularly effective for that individual.

## Data availability statement

The raw data supporting the conclusions of this article will be made available by the authors, without undue reservation.

## Author contributions

LQ, PY, and PB designed the experiments. LQ, PY, KC, KS, and PB wrote and edited the manuscript. PY, XS, and KC generated the organoids with GWI veteran hiPSC cells provided by BBRAIN. PY and KC performed the immunostaining and analysis. All authors have read and approved the final version of the manuscript.
