# Supplementary material for: Veteran-derived cerebral organoids display multifaceted pathological defects in studies on Gulf War Illness
Source: Front Cell Neurosci. 2022 Dec 23;16:979652. doi: 10.3389/fncel.2022.979652 (PMC9816432; doi:10.3389/fncel.2022.979652)
Supplement: Supplementary file 1 [file Data_Sheet_1.pdf]

Supplemental Tables

Tables 1-4. Organoids were generated from control and case GWI hiPSC lines. Between 5-10 organoids were fixed with paraformaldehyde and sectioned onto slides at 20 microns. The control line samples were taken from 3 different organoid 'batches' and the case line samples were taken from 5 different organoid 'batches'. 20x images were taken across all of the sections, excluding major rips or necrotic areas.

Table 1. GW toxicant regimen produces tau pathology. Tau13 and ps422 AFU levels were obtained for each sample and subsequently normalized to the vehicle per line. Included in the table is this normalized AFU, the percent change, the standard deviation, standard error, and *p* value. Both stains were statistically analyzed with Kruskal-Wallis test.

**Table 1**	Control Vehicle	Control C+D	Case Vehicle	Case C+D
Organoids Analyzed	~20	~20	~30	~30
Avg Tau13 AFU	1	0.7571	0.9114	1.685
Percent change	-24.29%		77.36%	
SD (Tau13)	0.4072	0.1714	0.3679	0.7505
SE (Tau13)	0.09876	0.0225	0.06831	0.1287
p Value (Tau13)	0.0657		*<0.0001*	
Avg ps422 AFU	1	1.062	1	1.325
Percent change	6.20%		32.50%	
SD (ps422)	0.5037	0.4834	0.4971	0.6587
SE (ps422)	0.1222	0.06348	0.09076	0.1164
p Value (ps422)	>0.9999		0.0493	

Table 2. GW toxicant regimen leads to a reduction in microtubule acetylation. ßIII and acetylated tubulin AFU levels were obtained for each image and then normalized to the vehicle per line. These datapoints were then analyzed for the ratio of ßIII and acetylated tubulin. Included in the table is this normalized AFU, the percent change, the standard deviation, standard error, and *p* value. Both stains were statistically analyzed with Kruskal-Wallis test. The ratio of ßIII and acetylated tubulin was analyzed with a one-way ANOVA.

**Table 2**	Control Vehicle	Control C+D	Case Vehicle	Case C+D
Organoids Analyzed	~20	~20	~30	~30
Avg ßIII AFU	1	1.078	1	0.9803
Percent change	7.80%		-1.97%	
SD (ßIII)	0.3818	0.471	0.4992	0.3999
SE (ßIII)	0.06749	0.08326	0.0927	0.07997
p Value (ßIII)	>0.9999		>0.9999	
Avg acetylated AFU	1	0.8822	1	0.6522
Percent change	-11.78%		-34.78%	
SD (acetylated)	0.7074	0.6081	0.6777	0.422
SE (acetylated)	0.125	0.1075	0.1281	0.08439
p Value (acetylated)	>0.9999		0.1412	
Avg ßIII/Acetylated ratio	1	0.8931	1	0.6805
Percent change	-10.69%		-31.95%	
SD (ratio)	0.4298	0.3032	0.4126	0.291
SE (ratio)	0.07598	1	0.07798	0.05821
p Value (ratio)	0.4255		*0.0039*	

Table 3. GWI toxicant regimen leads to upregulation of the reactive astrocyte marker GFAP. GFAP and S100ß AFU was generated for each image and then normalized to the vehicle per line. Included in the table is this normalized AFU, the percent change, the standard deviation, standard error, and *p* value. Both stains were statistically analyzed with Kruskal-Wallis test.

**Table 3**	Control Vehicle	Control C+D	Case Vehicle	Case C+D
Organoids Analyzed	~20	~20	~30	~30
Avg GFAP AFU	0.9697	1.319	1	1.405
Percent change	34.93%		40.50%	
SD (GFAP)	0.4162	0.5796	0.5203	0.837
SE (GFAP)	0.05561	0.06831	0.0561	0.09601
p Value (GFAP)	*0.0012*		*0.0014*	
Avg S100B AFU	1	1.403	1	0.9736
Percent change	40.30%		-2.64%	
SD (S100B)	0.5497	1.056	0.611	0.6149
SE (S100B)	0.08585	0.165	0.1097	0.09846
p Value (S100B)	0.4552		>0.9999	

Table 4. GWI toxicant regimen alters neurogenesis. Tile scans of the whole section were taken at 20x magnification and only sections that had no (or minimal) rips and little to no central necrosis were used for the analysis. The total Sox2 and NeuN counts were performed with the ImageJ automatic cell counter across 13-24 total sections from at least 3-5 different organoids. The percent of loop that is proliferative was determined by zooming in on neuroepithelial loops in the tile scans and measuring the area of the Sox2 proliferative zone and subtracting the lumen area. Multiple loops per section were used, hence the higher amount of datapoints. The spread of Sox2 cells associated with a neuroepithelial loop or non-associated with a neuroepithelial loops was determined by adding the total number of Sox2 cells in all the loops of a section and subtracting that from the total number of Sox2 cells. Finally, the number of loops per section area was found by dividing the total number of loops in a section by the total section area. The last three experiments had a lower number of datapoints because each section had to include at least one neuroepithelial loop. All of these datapoints were normalized to vehicle per line. Included in the table is this normalized AFU, the percent change, the standard deviation, standard error, and *p* value. All but the NeuN counts were statistically analyzed with Kruskal-Wallis test. The NeuN count was analyzed with a one-way ANOVA.

**Table 4**	Control Vehicle	Control C+D	Case Vehicle	Case C+D
Organoids Analyzed	<10	<10	<10	<10
Total Sox2 Count per Section Area	1	1.152	1.077	0.5393
Percent change	15.20%		-53.77%	
SD (Sox2 Count)	0.4809	0.6545	0.7525	0.2311
SE (Sox2 Count)	0.1133	0.1336	0.1536	0.06411
p Value (Sox2 Count	>0.9999		*0.0208*	
Total NeuN Count per Section Area	1	0.7184	1	4.1
Percent change	-28.16%		310.00%	
SD (NeuN Count)	0.7612	0.5174	0.6215	2.395
SE (NeuN Count)	0.2111	0.1435	0.1874	0.6913
p Value (NeuN Count)	0.8287		*<0.0001*	
Avg Proliferative Area	1.003	0.9852	1.002	0.9953

Percent change		-1.78%			-0.67%	
SD (Proliferative Area)	0.02063		0.03471	0.02225		0.03203
SE (Proliferative Area)	0.003704		0.006808	0.00266		0.005492
p Value (Proliferative Area)		0.0878			>0.9999	
Avg Sox2 cells outside proliferative area	1		0.9891	1		0.9156
Percent change		-1.09%			-8.44%	
SD (Sox2 cells outside proliferative area)	0.1545		0.2604	0.1173		0.2945
SE (Sox2 cells outside proliferative area)	0.05838		0.09208	0.05865		0.1113
p Value (Sox2 cells outside proliferative area)		0.952			>0.9999	
Avg Sox2 cells inside proliferative area	1		1.058	1		1.242
Percent change		5.80%			24.20%	

SD (Sox2 cells inside proliferative area)	0.8243		1.39	0.3364		0.8446
SE (Sox2 cells inside proliferative area)	0.3116		0.4914	0.1682		0.3192
p Value (Sox2 cells inside proliferative area)		0.9188			>0.9999	
Avg Sox2 cells outside proliferative area	0.9995		0.7595	1.002		0.8451
Percent change		-24.00%			-15.69%	
SD (Sox2 cells outside proliferative area)	0.8521		0.5202	1.087		0.7479
SE (Sox2 cells outside proliferative area)	0.3013		0.1839	0.4859		0.2827
p Value (Sox2 cells outside proliferative area)		>0.9999			>0.9999	
Avg neuroepithelial loops per section area	0.9995		0.7595	1.002		0.8451
Percent change		-24.00%			-15.69%	
SD (neuroepithelial loops)	0.8521		0.5202	1.087		0.7479
SE (neuroepithelial loops)	0.3013		0.1839	0.4859		0.2827
p Value (neuroepithelial loops)		>0.9999			>0.9999	
